# Fat mass and obesity-associated protein (FTO) mediates signal transducer and activator of transcription 3 (STAT3)-drived resistance of breast cancer to doxorubicin

**DOI:** 10.1080/21655979.2021.1924544

**Published:** 2021-06-02

**Authors:** Yan Wang, Zhiqiang Cheng, Jing Xu, Meina Lai, Liming Liu, Min Zuo, Lin Dang

**Affiliations:** a Department of Pathology, Shenzhen People’s Hospital, The Second Clinical Medical College of Jinan University, the First Affiliated Hospital of Southern University of Science and Technology, Shenzhen, China; b Department of Dermatology, Shenzhen People’s Hospital, The Second Clinical Medical College of Jinan University, the First Affiliated Hospital of Southern University of Science and Technology, Shenzhen, China

**Keywords:** FTO, Doxorubicin resistance, breast cancer, Stat3, Chemosensitivity

## Abstract

Excessive activation of signal transducer and activator of transcription 3 (STAT3) is implicated in breast cancer (BC) chemoresistance, but its underlying mechanism is not fully understood. There are STAT3 binding sites in fat mass and obesity-associated protein (FTO) promoter region, thus STAT3 may regulate the transcription of FTO. This study aimed to investigate the correlation between FTO and STAT3 in BC chemoresistance. Herein, FTO and STAT3 were highly expressed in doxorubicin-resistant BC (BC-DoxR) cells. CHIP assay verified the binding between STAT3 and FTO promoter in BC-DoxR cells. Dual luciferase reporter assay showed that FTO promoter activity was inhibited by S3I-201 (STAT3 inhibitor) but enhanced by epidermal growth factor (EGF, STAT3 activator) in BC-DoxR and BC cells. FTO mRNA and protein expression were suppressed by S3I-201 in BC-DoxR cells and EGF-stimulated BC cells. Notably, FTO regulated total N6-methyladenosine (m6A) levels in BC-DoxR and BC cells but could not affect STAT3 mRNA expression, indicating that FTO was not involved in the m6A modification of STAT3. However, FTO could activate STAT3 signaling in BC-DoxR and BC cells. Besides, FTO knockdown inhibited the doxorubicin resistance of BC-DoxR cells, while FTO overexpression enhanced the doxorubicin resistance and weakened the doxorubicin sensitivity of BC cells. Moreover, decreased doxorubicin resistance by STAT3 knockdown was abolished by FTO overexpression and decreased doxorubicin sensitivity by STAT3 overexpression was reversed by FTO knockdown, indicating that FTO was implicated in STAT3-mediated doxorubicin resistance and impairment of doxorubicin sensitivity of BC cells. Altogether, our findings provide a mechanism underlying BC doxorubicin resistance.

## Graphical abstract

**Figure:**
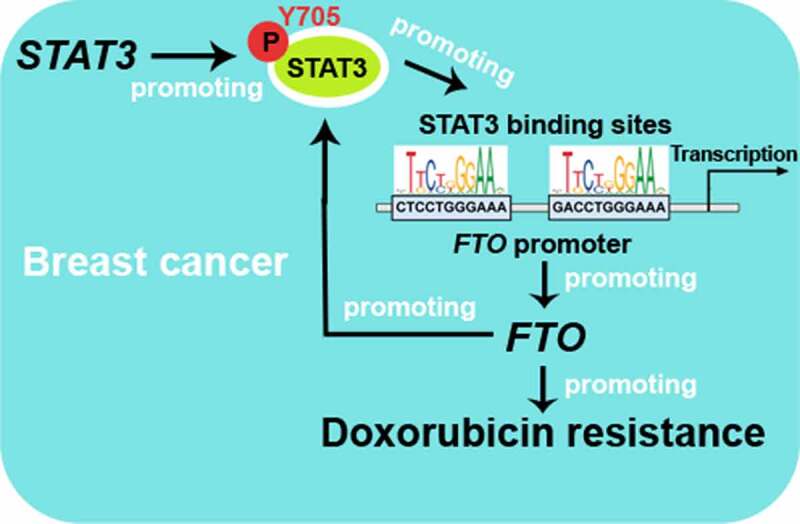

## Introduction

Breast cancer (BC) is the most commonly diagnosed cancer all over the world, which is responsible for about 11.7% of the total cancer incidence [1]. An estimated 2.3 million new global BC cases occurs in 2020, contributing to about 1 in 4 female cancer cases and 1 in 6 female cancer deaths [1]. BC is still the leading cause of cancer-related death in women worldwide [1]. Patients diagnosed with localized BC receive timely treatment, most of whom can have an excellent prognosis. The 5-year survival rate for BC patients diagnosed at Stage I is nearly 100%, but for those diagnosed at Stage IV, the 5-year survival rate is only 26% [2]. Chemotherapy is one of the most frequently used treatment strategies for BC. Furthermore, chemoresistance has become the significant constraint in successful BC treatment. Hence, a better understanding of the mechanisms underlying BC chemoresistance is of great significance.

Fat mass and obesity-associated protein (FTO), the first identified N6-methyladenosine (m6A) mRNA demethylase, has been well known for its association with an increased risk of obesity [3]. Accumulating evidence has shown that FTO contributes to the development and progression of cancers, including endometrial cancer [4], ovarian cancer [5], pancreatic cancer [6], lung squamous cell carcinoma [7], melanoma [8] and acute myeloid leukemia [9]. Previous studies have shown that FTO expression was up-regulated in the breast tissues of BC patients [10,11]. Furthermore, FTO has been implicated in regulating the proliferation, apoptosis and metastasis of BC cells [10]. These findings highlight the important role of FTO in the development of BC.

Signal transducer and activator of transcription 3 (STAT3) is a cytoplasmic signal transcription factor that mediates cytokine and growth factor signaling pathway [12]. Aberrantly activated STAT3 has been reported in various tumors, including renal tumors [13], esophageal squamous cell carcinoma [14], multiple myeloma [15], prostatic carcinomas [16] and BC [17]. Further activated STAT3 plays a pivotal role in carcinogenesis [18,19]. Excessive activation of STAT3 has been reported to be strongly associated with the chemoresistance of BC [20–22]. Emerging studies have revealed that FTO could promote STAT3 activation as well as inhibit its activation [23,24]. However, the relationship between FTO and STAT3 in BC remains unclear. As a transcription factor, STAT3 has been reported to promote chemoresistance of BC by binding to the promoter of an oncogene Midline2 [25]. It also needs further investigations whether STAT3 can bind to FTO promoter.

To investigate the correlation between FTO and STAT3 in BC chemoresistance and the role of STAT3-FTO in BC chemoresistance, doxorubicin-resistant BC (BC-DoxR) cells were collected to evaluate the FTO and STAT3 expression pattern. Then we explored the role of STAT3 in the transcription activity of FTO promoter and the expression of FTO in BC-DoxR and BC cells. Subsequently, the effect of FTO on the activation of STAT3 signaling in BC-DoxR and BC cells was also investigated. Finally, the function of STAT3-FTO circuit in doxorubicin resistance of BC cells and doxorubicin sensitivity of triple-negative BC (TNBC) cell were verified. This work manifested the molecular mechanism underlying the chemoresistance of BC cells.

## Methods

### Cell culture

Luminal A type BC cell line MCF-7 and two TNBC cell lines MDA-MB-231 and Hs578T were purchased from Procell Life Science &Technology Co.,Ltd. and iCell Bioscience Inc. (China). Cells were maintained at 37°C in a 5% CO_2_-humidified incubator (Heal Force, China). MCF-7 cells were cultured in Minimum Essential Medium (Gibco Life Technologies, USA) containing 10% fetal bovine serum (FBS, Every Green, China), MDA-MB-231 cells in Leibovitz’s L-15 Medium (Procell) containing 10% FBS and Hs578T in Dulbecco’s modified eagle medium (Gibco Life Technologies) containing 10% FBS (Every Green).

### Induction of doxorubicin resistance

The BC-DoxR cells were generated by exposing the parental cells to different concentrations of doxorubicin (10–100 nM, Aladdin, China) as previously described [26]. Cells were considered doxorubicin-resistant after surviving 7 passages. Then the dxorubicin-resistant MCF-7 and MDA-MB-231 cells were cultured in Minimum Essential Medium (Gibco Life Technologies) and Leibovitz’s L-15 Medium (Procell) containing 10% FBS (Every Green) at 37°C with 5% CO_2_, respectively.

### Cell treatment and transfection

To obtain the half-maximal (50%) inhibitory concentration (IC50) value of doxorubicin in MCF-7/MDA-MB-231, MCF-7-DoxR/MDA-MB-231-DoxR and Hs578T cells, these cells were exposed to different levels of doxorubicin (0.01–10 μM) for 24 h in a humidified atmosphere at 37°C with 5% CO_2_. To verify the role of STAT3 in the expression of FTO, BC cells were treated with 5 or 10 ng/ml epidermal growth factor (EGF, Sino Biological, China) to promote STAT3 activation and 100 μM selective STAT3 inhibitor S3I-201 (MeilunBio, China) to inhibit STAT3 DNA-binding activity for 24 h, while BC-DoxR cells were treated with 30 or 100 μM S3I-201 for 24 h.

BC-DoxR cells or their parental cells were transfected with FTO shRNA (shFTO), plasmids pcDNA3.1 (Invitrogen, USA) containing the *FTO* gene or their negative control (NC shRNA, shNC; empty vector, OE-NC). Seventy-two h post-transfection, the cells were used for further analyses. BC-DoxR cells or their parental cells were transfected with FTO siRNA (siFTO; siFTO-1, sense 5ʹ-CCACGUUGAUAAGGCACAATT-3ʹ, antisense 5ʹ-UUGUGCCUUAUCAACGUGGTT-3ʹ; siFTO-2, sense 5ʹ-GGGUGUGAUAAGUGUGGAGTT-3ʹ, antisense 5ʹ-CUCCACACUUAUCACACCCTT-3ʹ), plasmids pcDNA3.1 (Invitrogen, USA) containing the *FTO* gene or their negative control (NC siRNA, siNC; OE-NC) and co-transfected with STAT3 siRNA (siSTAT3; sense 5ʹ-CCACUUUGGUGUUUCAUAATT-3ʹ, antisense 5ʹ-UUAUGAAACACCAAAGUGGTT-3ʹ) and plasmids pcDNA3.1 containing the *FTO* gene using Lipofectamine 2000 (Invitrogen). Forty-eight h post-transfection, the cells were exposed to doxorubicin (half of IC50) for 24 h. Hs578T cells were transfected with plasmid pcDNA3.1 containing the *FTO* gene or its negative control (OE-NC) and co-transfected with plasmids pcDNA3.1 containing the *STAT3* gene and siFTO-1 using Lipofectamine 2000 (Invitrogen). Forty-eight h post-transfection, Hs578T cells were exposed to doxorubicin (half of IC50) for 24 h.

### Cell counting kit-8 (CCK-8) assay

Cell proliferation was measured using the CCK-8 assay Kit (Sigma-Aldrich, USA). Cells were seeded into 96-well culture plates at a density of 4 × 10^3^ cells per well. For detection, cells were incubated with 10 µL CCK-8 solution for 1 h at 37°C in a 5% CO_2_-humidified incubator, then the absorbance was measured at 450 nm using a microplate reader (BioTek Instruments, USA).

### Real-time quantitative PCR (RT-qPCR)

The mRNA expression level of genes was analyzed using RT-qPCR. Total RNA was extracted from cells using the RNApure High-purity Total RNA Rapid Extraction Kit (BioTeke, China). Complementary DNA was synthesized from the total RNA samples by reverse-transcribing with reverse transcriptase M-MLV (Takara), oligo (dT)15 and random primers (Genscript, China). Next, RT-qPCR was carried out using SYBR Green (BioTeke) and Taq™ HS Perfect Mix (Takara). The sequence of primers used in RT-qPCR was presented in Table 1. The mRNA expression level of β-actin was used as an endogenous control. The results were analyzed using the 2^−ΔΔCt^ method.

**Table 1.** The sequence of primers used in RT-qPCR

	Primers	
	Type	Sequence (5′-3′)
FTO	Forward	GAACACCAGGCTCTTTACG
	Reverse	ATGAACCCATCCCAACC
STAT3	Forward	TGGAGAAGGACATCAGCGGT
	Reverse	TGGTCTTCAGGTATGGGGCA
β-actin	Forward	CACTGTGCCCATCTACGAGG
	Reverse	TAATGTCACGCACGATTTCC

### Western blotting analysis

Cells were harvested and lysed with RIPA Lysis Buffer (beyotime, China) containing 1% protease inhibitor  PMSF (beyotime). The cell lysates were separated by SDS-PAGE and transferred to PVDF membranes (Thermo Fisher Scientific, USA). After blocking with 5% (M/V) BSA, the membrane was incubated with primary antibodies including anti-FTO (1:1000, ABclonal, China), anti-phospho STAT3 (Tyr705) (p-STAT3, 1:500, affinity, China), anti-STAT3 (1:500, affinity), anti-cleaved poly (ADP-ribose) polymerase (PARP) (1:500, CST, USA), anti-cleaved caspase-3 (1:500, affinity) and anti-β-actin (1:2000, proteintech, China) at 4°C overnight, followed by incubation with HRP-conjugated Goat anti-Rabbit or Goat anti-Mouse IgG secondary antibodies (1:10,000, proteintech) at 37°C for 40 min. The protein bands were quantified using the Gel-Pro-Analyzer software. β-actin was served as an endogenous control.

### Chromatin immunoprecipitation (CHIP) assay

CHIP assay was performed using the CHIP Assay Kit (Wanleibio, China) according to the manufacturer’s protocol. The immunoprecipitated DNA was purified using the PCR Clean-Up Kit (Wanleibio) and then amplified with 2× Power Taq PCR MasterMix (Bioteke) and primers (FTO promoter forward: 5ʹ-ACCTCCACCCACCCTCAT-3ʹ, reverse: 5ʹ-GCCACGGGATTTAGCACAG-3ʹ, Genscript). The PCR-amplified product of the DNA samples was confirmed using 1.5% agarose gel electrophoresis.

### Dual luciferase reporter assay

The fragment of FTO promoter containing the predicted STAT3-binding sites was cloned into pGL3 reporter vectors (Promega Corporation, USA). The MCF-7-DoxR cells and MCF-7 cells were transfected with the reporter vector containing the fragment of FTO promoter or empty reporter vector using Lipofectamine™ 2000 Reagent (Invitrogen). Forty-eight h post-transfection, the transfected MCF-7-DoxR cells were treated with 100 μM S3I-201 for 6 or 24 h, while the transfected MCF-7 cells were treated with 10 ng/ml EGF for 6 or 24 h. The ratio of firefly to Renilla luciferase luminescence was calculated and normalized to the ratio of the empty reporter group.

### Detection of m6A RNA methylation

The m6A RNA methylation was measured using the M6A RNA Methylation Quantification Kit (EpiQuik, USA) according to the manufacturer’s instruction.

### Cell apoptosis assay

The transfected BC-DoxR cells and BC cells were collected and washed twice with PBS. Next, the cells were stained using Annexin V-PI Apoptosis Detection Kit (Beyotime, China) according to the manufacturer’s instruction and then detected using a NovoCyte Flow Cytometer (Acea Biosciences, USA). The cell apoptotic rate was calculated as follows: Apoptotic cells (%) = early apoptotic cells (Annexin V^+^/PI^−^, %) + late apoptotic cells (Annexin V^+^/PI^+^, %).

### Statistical analysis

All data were shown as mean ± standard deviation. Each experiment contained at least three replicates. Graphpad prism 8 was used to analyze data and make graphs. We used Student’s t-test to analyze differences between two groups and ordinary one-way ANOVA combined with Tukey’s multiple comparison test to analyze differences among three or more groups, and a P value less than 0.05 was considered statistically significant.

## Results

### Increased expression of FTO and STAT3 in BC-DoxR cells

**Figure 1.**
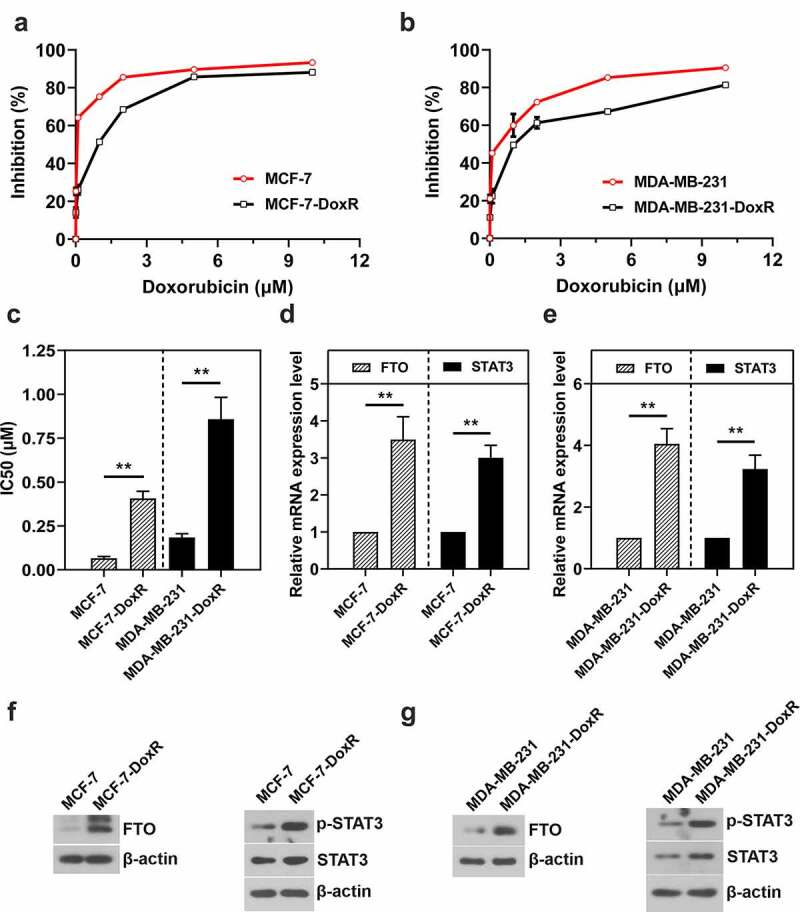
**FTO and STAT3 expression in doxorubicin-resistant BC cells**. a, b The sensitivity of doxorubicin-resistant MCF-7 and MDA-MB-231 cells and their parental cells to doxorubicin. c The IC50 value of doxorubicin against doxorubicin-resistant MCF-7 and MDA-MB-231 cells and their parental cells. d, e The mRNA level of FTO and STAT3 in doxorubicin-resistant BC cells and their parental cells. f, g The protein expression of FTO, p-STAT3 and STAT3 in doxorubicin-resistant BC cells and their parental cells. **p < 0.01. MCF-7-DoxR, doxorubicin-resistant MCF-7; MDA-MB-231-DoxR, doxorubicin-resistant MDA-MB-231; IC50, half-maximal (50%) inhibitory concentration; p-STAT3, phosopho-STAT3^Tyr705^

To investigate the expression of FTO and STAT3 in BC-DoxR cells, two commercially available BC cell lines including MCF-7 and MDA-MB-231 were used to develop doxorubicin-resistant population MCF-7-DoxR and MDA-MB-231-DoxR. CCK-8 assay was carried out to determine the resistance of BC cells to doxorubicin. As shown in Figure 1(a,b), the resistant ability of MCF-7-DoxR and MDA-MB-231-DoxR cells to doxorubicin was significantly higher compared with their parental cells. MCF-7-DoxR (IC50 value: 0.41 μM) and MDA-MB-231-DoxR (IC50 value: 0.86 μM) cells were about 6.19 and 4.66 times more resistant than their parental cells (IC50 value-MCF-7: 0.07 μM; IC 50 value-MDA-MB-231: 0.18 μM) (Figure 1(c)). RT-qPCR and western blotting were performed to measure FTO and STAT3 mRNA and protein expressions, respectively. FTO and STAT3 mRNA expression levels in MCF-7-DoxR and MDA-MB-231-DoxR cells were significantly up-regulated compared with their parental cells (Figure 1(d,e)). Moreover, FTO and p-STAT3 protein expression in MCF-7-DoxR and MDA-MB-231-DoxR cells were also increased (Figure 1 (f,g)).

**STAT3 binds to FTO promoter and enhances the activity of FTO promoter and FTO expression in BC-DoxR and BC cells**

**Figure 2.**
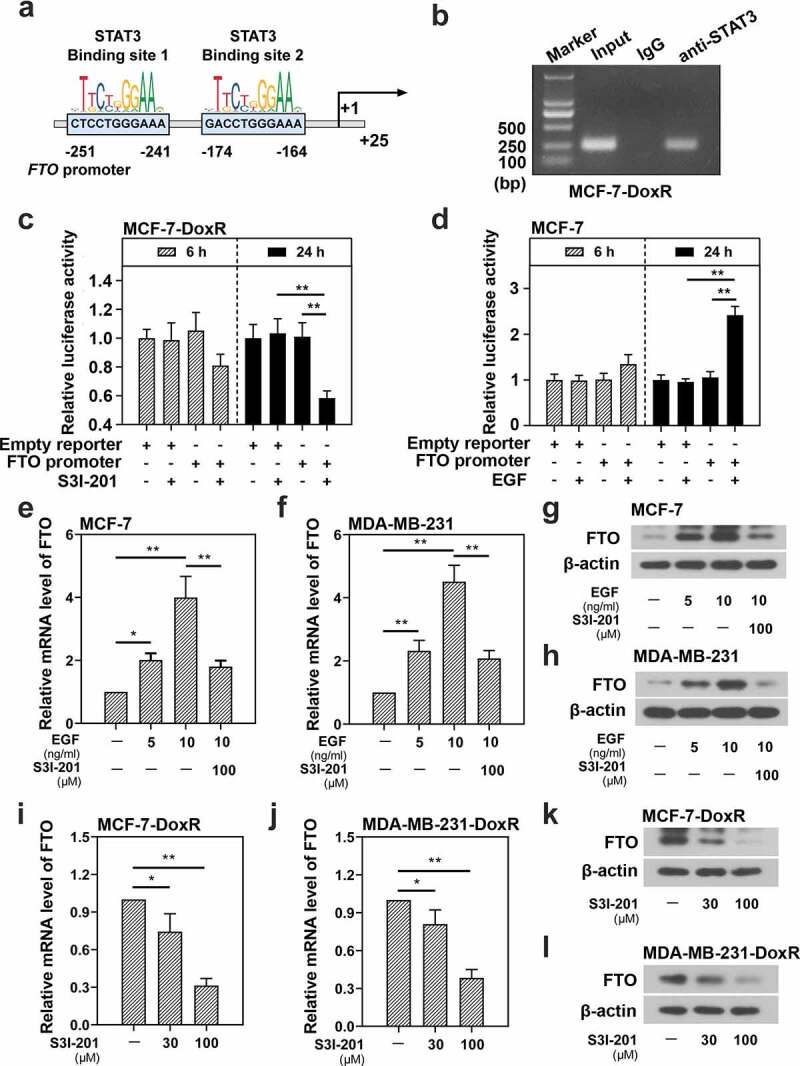
**Effect of STAT3 on the transcriptional activity of FTO promoter and the expression of FTO in doxorubicin-resistant BC cells and their parental cells**. a The binding sites of STAT3 in FTO promoter predicted by JASPAR. b CHIP assay confirmed the binding between FTO promoter and STAT3 in doxorubicin-resistant MCF-7 cells. c, d Dual luciferase reporter assay confirmed that the transcriptional activity of FTO promoter was inhibited by S3I-201 but enhanced by EGF in doxorubicin-resistant MCF-7 cells and MCF-7 cells. e-l The mRNA and protein expression of FTO was evaluated using RT-qPCR and western blotting. *p < 0.05, **p < 0.01. MCF-7-DoxR, doxorubicin-resistant MCF-7; MDA-MB-231-DoxR, doxorubicin-resistant MDA-MB-231; EGF, epidermal growth factor

To explore the relationship between STAT3 and FTO, the JASPAR database (http://jaspar.genereg.net/) was used to predict whether the binding sites of STAT3 existed in FTO promoter region (Figure 2(a)). CHIP assay was performed to determine the relationship between STAT3 and FTO promoter. It was confirmed that STAT3 could bind to the FTO promoter in MCF-7-DoxR cells (Figure 2(b)). Subsequently, the effect of STAT3 inhibitor and activator on the activity of FTO promoter was analyzed by dual luciferase reporter assay. The efficiency of STAT3 inhibitor S3I-201 has been widely reported and that of STAT3 activator EGF on MCF-7 cells was shown in Figure S1A. S3I-201 significantly decreased the relative luciferase activity of FTO promoter in MCF-7-DoxR cells (Figure 2(c)), while EGF increased the relative luciferase activity of FTO promoter in MCF-7 cells (Figure 2(d)). The results indicated that STAT3 could enhance the activity of FTO promoter in BC-DoxR and BC cells. Then we detected the expression of FTO in BC-DoxR and BC cells after stimulation with EGF or/and S3I-201 using RT-qPCR and western blotting. The efficiency of STAT3 activator EGF on MDA-MB-231 cells was shown in Figure S1B. EGF significantly up-regulated the mRNA and protein expression of FTO in BC cells, yet further S3I-201 stimulation down-regulated the EGF-induced mRNA and protein expression of FTO (Figure 2(e-h)). Moreover, S3I-201 stimulation led to down-regulation in the mRNA and protein expression of FTO in BC-DoxR cells (Figure 2(i-l)). These results illustrated that STAT3 positively regulated the expression of FTO in BC-DoxR and their parental cells.

### FTO positively regulates the activation of STAT3 signaling in BC-DoxR and BC cells

**Figure 3.**
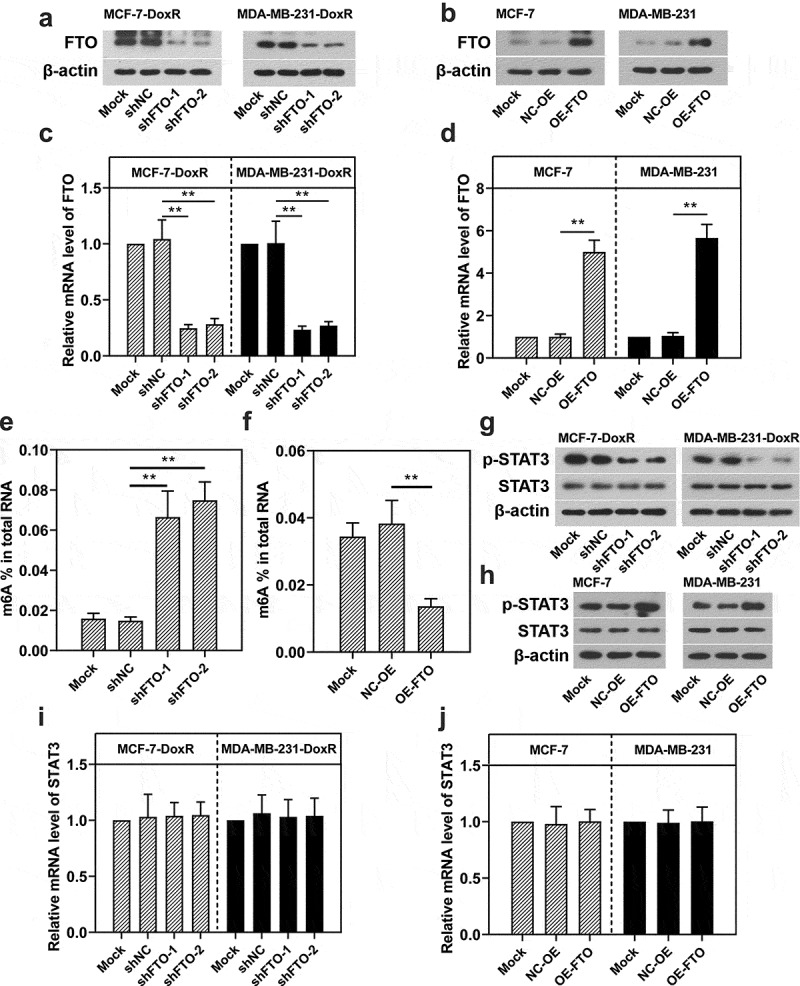
**Effect of FTO on the activation of STAT3 signaling in doxorubicin-resistant BC cells and their parental cells**. Doxorubicin-resistant BC cells and their parental cells were transfected with FTO shRNAs and FTO overexpression plasmids for 72 h, respectively. a-d The protein and mRNA expression of FTO in transfected and untransfected cells. e, f Total m6A level in transfected and untransfected cells. g-j The protein expression of p-STAT3 and STAT3 and the mRNA expression of STAT3 in transfected and untransfected cells. **p < 0.01. MCF-7-DoxR, doxorubicin-resistant MCF-7; MDA-MB-231-DoxR, doxorubicin-resistant MDA-MB-231; shNC, negative control shRNA; shFTO, FTO shRNA; NC-OE, empty vector; OE-FTO, FTO overexpression; p-STAT3, phosopho-STAT3^Tyr705^; m6A, N6-methyladenosine

To determine the effect of FTO on the STAT3 expression, two shRNAs for FTO were transfected into BC-DoxR cells, while plasmids overexpressing FTO were transfected into BC cells. As shown in Figure 3(a-d), the introduction of FTO shRNAs weakened the mRNA and protein expression of FTO in BC-DoxR cells and plasmids overexpressing FTO increased the mRNA and protein expression of FTO in BC cells. In addition, FTO silencing was observed to increase the m6A level in MCF-7-DoxR cells (Figure 3(e)). On the contrary, FTO overexpression decreased the m6A level in MCF-7 cells (Figure 3(f)). It was indicated that FTO was involved in the mRNA m6A modification in BC-DoxR and their parental cells. The SRAMP database (http://www.cuilab.cn/sramp) predicted that there were several possible m6A modification sites in STAT3. To verify the interaction between FTO and STAT3, RT-qPCR and western blotting were performed to measure STAT3 mRNA expression and p-STAT3 and STAT3 protein expression. FTO silencing significantly decreased the protein expression of p-STAT3 in BC-DoxR cells, while FTO overexpression increased its expressions in BC cells (Figure 3(g,h)), respectively. Notably, the mRNA expression of STAT3 in BC-DoxR cells and their parental cells was not significantly influenced by FTO silencing and overexpression (Figure 3(i,j)). These results indicated that FTO was able to activate STAT3 signaling in BC-DoxR and BC cells. Nevertheless, FTO did not contribute to the m6A modification of STAT3 in BC-DoxR and BC cells.

### STAT3 facilitates the resistance of BC cells to doxorubicin via FTO

**Figure 4.**
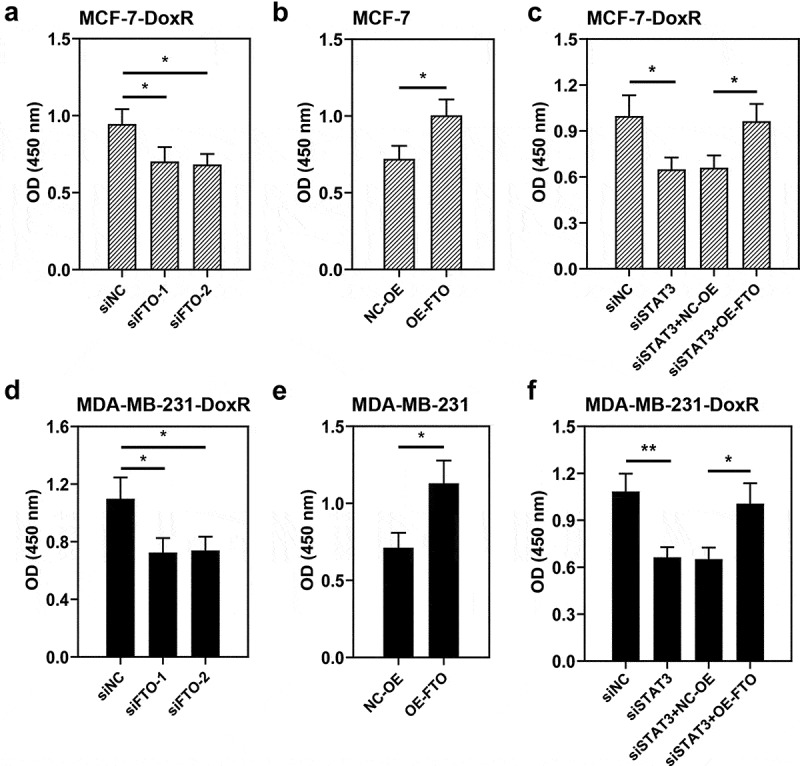
**Role of STAT3-FTO in the doxorubicin-induced loss of BC cell viability**. Doxorubicin-resistant BC or BC cells were transfected with FTO siRNAs or FTO overexpression plasmids and co-transfected with FTO siRNA and FTO overexpression plasmids for 48 h and then exposed to doxorubicin for 24 h. a-f Cell viability was measured by CCK-8 assay. *p < 0.05, **p < 0.01. MCF-7-DoxR, doxorubicin-resistant MCF-7; MDA-MB-231-DoxR, doxorubicin-resistant MDA-MB-231; siNC, negative control siRNA; siFTO, FTO siRNA; NC-OE, empty vector; OE-FTO, FTO overexpression; OD, optical density

**Figure 5.**
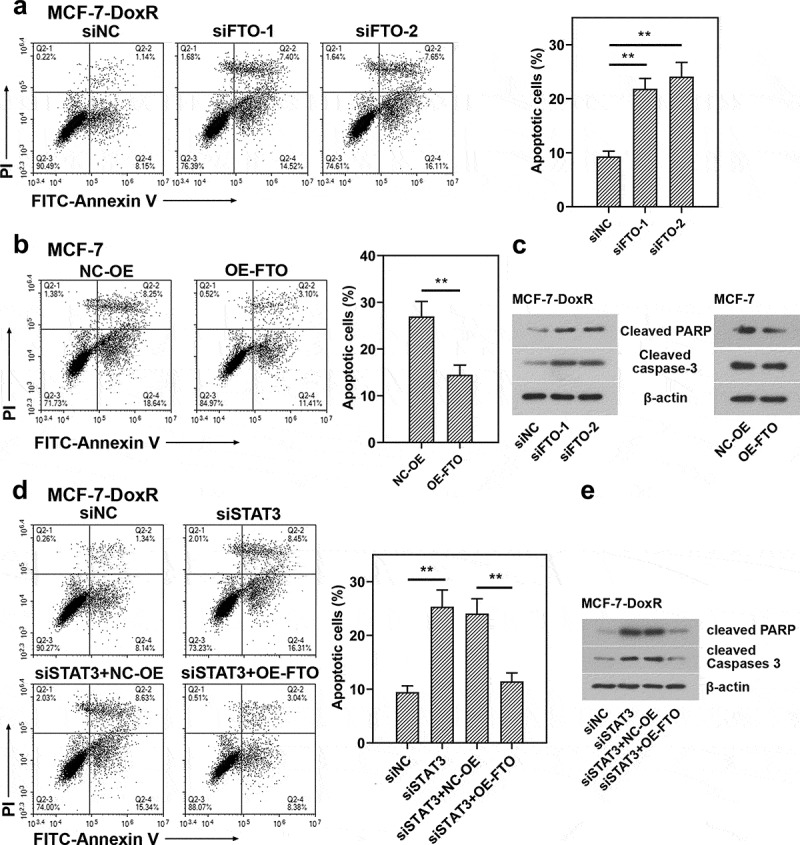
**Role of STAT3-FTO in the doxorubicin-induced apoptosis of MCF-7 cells**. Doxorubicin-resistant MCF-7 or MCF-7 cells were transfected with FTO siRNAs or FTO overexpression plasmids and co-transfected with FTO siRNA and FTO overexpression plasmids for 48 h and then exposed to doxorubicin for 24 h. a, b and d Apoptotic MCF-7 cells were detected by Annexin V/PI staining. c, e Expressions of cleaved PARP and cleaved caspase-3 in doxorubicin-resistant MCF-7 and MCF-7 cells. **p < 0.01. MCF-7-DoxR, doxorubicin-resistant MCF-7; siNC, negative control siRNA; siFTO, FTO siRNA; NC-OE, empty vector; OE-FTO, FTO overexpression; PI, propidium iodide; PARP, poly (ADP-ribose) polymerase

**Figure 6.**
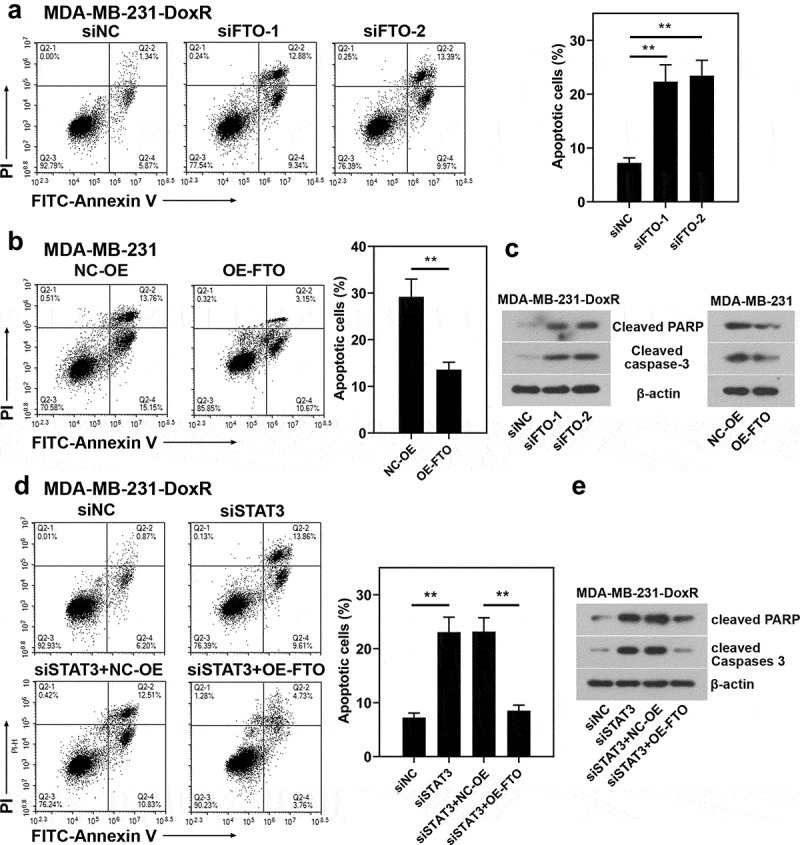
**Role of STAT3-FTO in the doxorubicin-induced apoptosis of MDA-MB-231 cells**. Doxorubicin-resistant MDA-MB-231 or MDA-MB-231 cells were transfected with FTO siRNAs or FTO overexpression plasmids and co-transfected with FTO siRNA and FTO overexpression plasmids for 48 h and then exposed to doxorubicin for 24 h. a, b and d Apoptotic MDA-MB-231 cells were detected by Annexin V/PI staining. c, e Expressions of cleaved PARP and cleaved caspase-3 in doxorubicin-resistant MDA-MB-231 and MDA-MB-231 cells. **p < 0.01. MDA-MB-231-DoxR, doxorubicin-resistant MDA-MB-231; siNC, negative control siRNA; siFTO, FTO siRNA; NC-OE, empty vector; OE-FTO, FTO overexpression; PI, propidium iodide; PARP, poly (ADP-ribose) polymerase

To evaluate the role of FTO-STAT3 in the resistance of BC cells to doxorubicin, cells were transfected with FTO siRNAs or plasmids overexpressing FTO and co-transfected with STAT3 siRNA and plasmids overexpressing FTO. Then the transfected and untransfected BC-DoxR and BC cells were exposed to doxorubicin. Cell viability was measured using CCK-8 assay and cell apoptosis was detected using Annexin V-PI staining. The apoptosis-related proteins including cleaved PARP and cleaved caspase-3 were analyzed using western blotting. Results showed that doxorubicin exposure resulted in decreases in cell viability and increases in apoptotic cells and expressions of cleaved PARP and cleaved caspase-3 in BC and BC-DoxR cells (Figure S2-S4). FTO siRNAs weakened FTO expression in BC-DoxR cells and plasmids overexpressing FTO increased FTO expression in BC cells (Figure S5A-D). The results of CCK-8 assay showed that the viability of BC cells exposed to doxorubicin was decreased by FTO silencing but increased by FTO overexpression (Figure 4(a,b,d,e). The results of Annexin V-PI staining and western blotting exhibited that doxorubicin-induced BC cell apoptosis and expressions of cleaved PARP and cleaved caspase-3 were promoted by FTO silencing yet inhibited by FTO overexpression (Figure 5(a-c) and Figure 6(a-c)). The findings suggested that FTO facilitated the resistance of BC cells to doxorubicin. In addition, STAT3 siRNA weakened the expression of STAT3 and p-STAT3 and plasmids overexpressing FTO increased the expression of p-STAT3 in BC-DoxR cells (Figure S6). The results displayed that FTO overexpression reversed the loss of BC cell viability and promotion in cell apoptosis and expressions of cleaved PARP and cleaved caspase-3 induced by doxorubicin exposure and STAT3 silencing (Figure 4(c,f), Figure 5(d,e) and Figure 6(d,e)). The results revealed that STAT3 facilitates the resistance of BC cells to doxorubicin via FTO.

### STAT3 impairs the sensitivity of TNBC cells to doxorubicin via FTO

**Figure 7.**
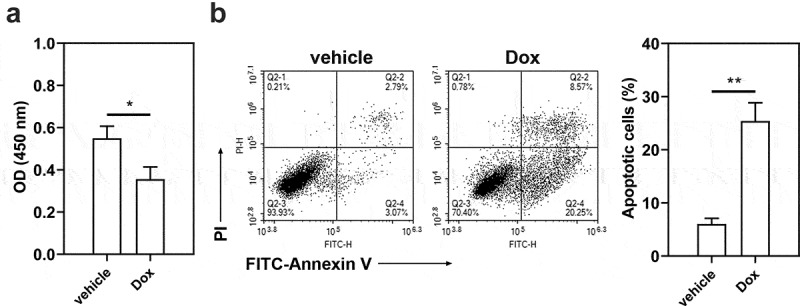
**Effect of doxorubicin on the viability and apoptosis of Hs578T cells**. Hs578T cells were exposed to doxorubicin for 24 h. a Cell viability was measured by CCK-8 assay. b Apoptotic Hs578T cells were detected by Annexin V/PI staining. *p < 0.05, **p < 0.01. Dox, doxorubicin; OD, optical density; PI, propidium iodide

**Figure 8.**
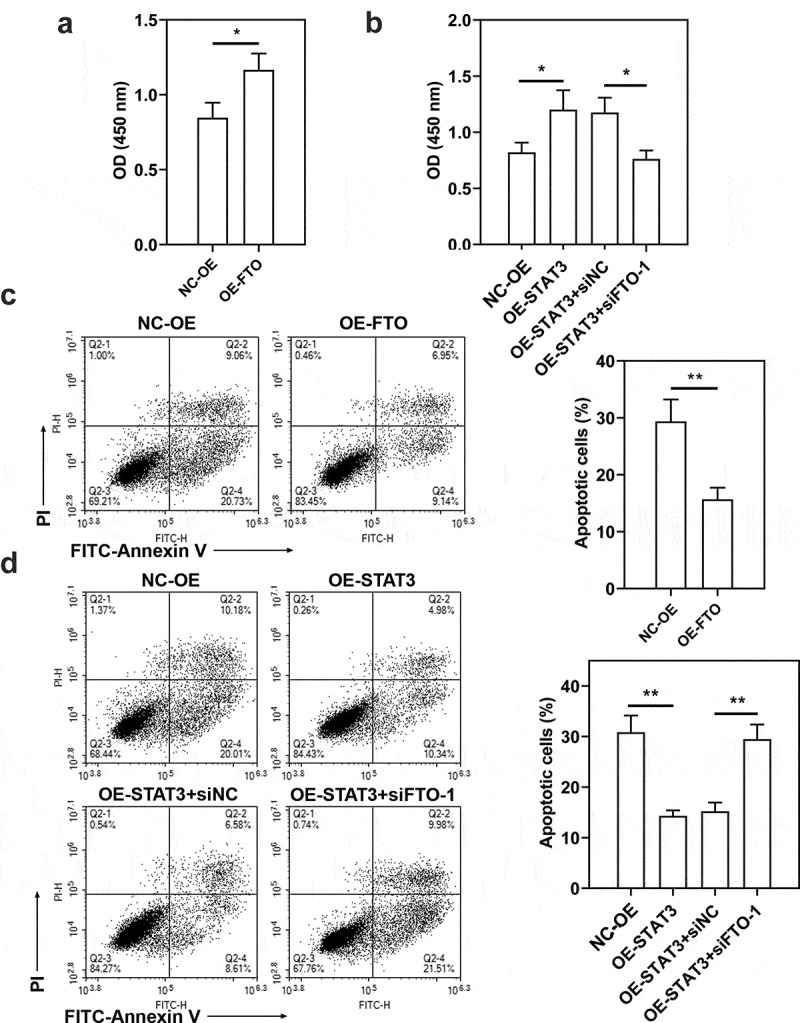
**Role of STAT3-FTO in the doxorubicin-induced viability loss and apoptosis of Hs578T cells**. Hs578T cells were transfected with FTO overexpression plasmids and co-transfected with STAT3 overexpression plasmids and FTO siRNA-1 for 48 h and then exposed to doxorubicin for 24 h. a, b Cell viability was measured by CCK-8 assay. c, d Apoptotic Hs578T cells were detected by Annexin V/PI staining. *p < 0.05, **p < 0.01. Dox, doxorubicin; OD, optical density; PI, propidium iodide; NC-OE, empty vector; OE-FTO, FTO overexpression; OE-STAT3, STAT3 overexpression; siNC, negative control siRNA; siFTO-1, FTO siRNA-1

To confirm the role of STAT3-FTO in the sensitivity of TNBC cells to doxorubicin, Hs578T cells were exposed to different levels of doxorubicin (0.01–10 μM) to evaluate IC50 value (Figure S7, IC50 value: 4.57 μM). Then Hs578T cells were exposed to doxorubicin for 24 h. Doxorubicin exposure decreased cell viability and increased cell apoptosis (Figure 7(a,b)). FTO overexpression led to increases in cell viability and decreases in cell apoptosis (Figure 8(a,c)), and STAT3 overexpression exhibited a similar effect (Figure 8(b,d)). However, FTO knockdown reversed the increases in cell viability and decreases in cell apoptosis induced by STAT3 overexpression (Figure 8(b,d)). These results implied that STAT3 impairs the sensitivity of TNBC cells to doxorubicin via FTO.

## Discussion

Doxorubicin, a type of anthracycline, has been widely used as a classical chemotherapy drug in the treatment of many cancers, including BC [27]. However, resistance to doxorubicin is by far one of the most important obstacles on the treatment of BC, which can diminish the efficiency of doxorubicin and results in tumor growth [28]. Therefore, we discussed the mechanisms underlying the resistance of BC to doxorubicin in the present study. Early reports showed that there was a close association between FTO and the occurrence and development of obesity and type 2 diabetes [29,30]. Later, FTO was found to be highly expressed in cancers and correlate with the risk of various human cancers [31,32,33]. FTO has been involved in regulating multiple aspects of cancer biology, including cell proliferation, migration, invasion and apoptosis [6,34,35]. However, FTO is a double-edged sword in regulating the chemoradiotherapy sensitivity of cancer cells. On one hand, FTO overexpression inhibited cisplatin and irradiation-induced loss of cervical squamous cell carcinoma cell viability but FTO inhibitor MA2 increased the chemosensitivity of cervical squamous cell carcinoma cells [36]. Moreover, depletion of FTO expression by shRNA enhanced melanoma cell sensitivity to anti-PD-1 antibody [8]. On the other hand, up-regulated FTO expression sensitized leukemia cells to R-2-hydroxyglutarate [37]. The oncogenic potential of FTO in BC has also been recognized in previous studies, while its role in the chemotherapy sensitivity of BC remains unclear. In the present study, FTO mRNA and protein expression was higher in less doxorubicin-sensitive BC cells. Hence, it was speculated that FTO might contribute to the doxorubicin resistance of BC cells.

Activation of STAT3 via Y705-phosphorylation broadly exists in cancers, and its tumorigenic role has been widely recognized. Since STAT3 was discovered in 1994, most studies have mainly focused on its potential in the survival, proliferation, angiogenesis, migration, invasion and immune suppression of cancer cells [38]. In addition, STAT3 also plays a critical role in the chemoresistance of BC cells. Suppression of STAT3 activation by drugs has been confirmed to promote the chemosensitivity of BC cells [39]. Consistent with prior studies, p-STAT3 was highly expressed in less doxorubicin-sensitive BC cells in the present study, indicating that STAT3 was activated in BC-DoxR cells. Multiple types of cytokine including fibroblast growth factor, interleukin 6 and EGF can activate Janus kinases to trigger STAT3 phosphorylation by binding their corresponding receptors and S3I-201 shows potent inhibition of STAT3 DNA-binding activity [40,41]. Hence, EGF and S3I-201 were chosen to promote and inhibit STAT3 activation in this study. The phosphorylated STAT3 concentrates into nucleus and further binds to the promoter region of target genes to activate transcription [42]. JASPAR database showed that several STAT3-binding sites existed in the promoter region of FTO. In the present study, STAT3 was observed to bind to the promoter region of FTO, increase the activity of FTO promoter and positively regulate FTO mRNA and protein expression in BC and BC-DoxR cells. Therefore, the up-regulation of FTO expression in BC-DoxR cells may be attributed to the activation of STAT3.

FTO plays an oncogenic role through an m6A-dependent mechanism. FTO has been involved in the tumor progression of breast cancer via suppressing BCL2 interacting protein 3 in an m6A-dependent way [10]. SRAMP predicted potential m6A sites in STAT3. It was speculated that FTO might regulate the m6A modification of STAT3 to affect its expression. In the present study, in spite of significant changes in m6A levels, FTO silencing and overexpression could not affect STAT3 mRNA expression in BC and BC-DoxR cells, indicating that FTO was not implicated in the m6A modification of STAT3. FTO has been reported to regulate the phosphorylation of STAT3, and FTO knockdown decreased the p-STAT3 expression in porcine preadipocytes [24]. On the contrary, FTO overexpression in the liver of mice inhibited p-STAT3 expression [23]. The present study showed that the phosphorylation of STAT3 was inhibited by FTO silencing but promoted by FTO overexpression in BC-DoxR and BC cells, which indicated that FTO activated STAT3 signaling. It has been reported that knockdown of FTO might inhibit p-STAT3 expression by suppressing JAK2 mRNA level through influencing the m6A level of JAK2 [24]. Therefore, the phosphorylation of STAT3 regulated by FTO might be attributed to JAK2, which will be confirmed in our future work.

Subsequently, we further validated the role of STAT3-FTO in the doxorubicin resistance of BC cells. In the present study, FTO overexpression inhibited doxorubicin-induced loss of BC cell viability and BC cell apoptosis, indicating that FTO overexpression enhanced the doxorubicin resistance of BC cells. Contrarily, FTO knockdown decreased the doxorubicin resistance of BC-DoxR cells. In addition, STAT3 knockdown also inhibited the doxorubicin resistance of BC cells, while further FTO overexpression reversed STAT3 knockdown-induced decreases of doxorubicin resistance of BC cells, suggesting that STAT3 augmented the doxorubicin resistance of BC cells via FTO. Chemotherapy remains the standard of care for TNBC treatment, thus the role of STAT3-FTO circuit in the doxorubicin sensitivity of TNBC cells was verified. We found that FTO overexpression and STAT3 overexpression lowered the doxorubicin sensitivity of TNBC cells. However, FTO silencing reversed the inhibition of doxorubicin sensitivity of BC cells induced by STAT3 overexpression, suggesting that STAT3 impaired the doxorubicin sensitivity of TNBC cells via FTO.

There is a limitation in the present study. MCF-7 is a luminal A-type breast cancer cell line. Compared with chemotherapy, endocrine therapy is a more effective treatment strategy for luminal A-type breast cancer. Hence, a luminal A-type breast cancer cell line might not be the best candidate for the studies about chemotherapy.

## Conclusion

In summary, up-regulated FTO and STAT3 expressions were found in doxorubicin-resistant BC cells. STAT3 could bind to FTO promoter and increase the activity of FTO promoter to positively regulate FTO expression. Furthermore, FTO was also able to activate STAT3 signaling. Besides, FTO was involved in STAT3-mediated doxorubicin resistance of BC cells and impairment in doxorubicin sensitivity of TNBC cells. Altogether, our findings provide a mechanism underlying the resistance of BC cells to doxorubicin.

## Supplementary Material

Supplemental Material

## Data Availability

The datasets used in the present study are available from the corresponding author on reasonable request.

## Highlights

1. FTO and STAT3 were highly expressed in doxorubicin-resistant BC (BC-DoxR) cells.

2. STAT3 could bind to FTO promoter in BC-DoxR cells.

3. STAT3 positively regulated FTO expression in BC-DoxR cells.

4. FTO did not regulate the m6A modification of STAT3 but activated STAT3 signaling.

5. FTO was implicated in STAT3-mediated doxorubicin resistance of BC cells.
