# Supplementary material for: Fat mass and obesity-associated protein (FTO) mediates signal transducer and activator of transcription 3 (STAT3)-drived resistance of breast cancer to doxorubicin
Source: Bioengineered. 2021 Jun 2;12(1):1874–89. doi: 10.1080/21655979.2021.1924544 (PMC8806322; doi:10.1080/21655979.2021.1924544)
Supplement: Supplemental Material [file KBIE_A_1924544_SM7492.zip › Supplementary materials.docx]

**Figure S1 Effect of EGF on the p-STAT3 and STAT3 expression in BC cells.** A The p-STAT3 and STAT3 expression in MCF-cells. B The p-STAT3 and STAT3 expression in MDA-MB-231 cells. EGF, epidermal growth factor; p-STAT3, phosopho-STAT3^Tyr705^.

**Figure S2 Effect of doxorubicin on the viability of doxorubicin-resistant BC cells and their parental cells.** A, B The viability loss of doxorubicin-resistant BC cells by doxorubicin was less than that of BC cells (doxorubicin concentration: half of IC50 value of BC cells). C, D The viability of doxorubicin-resistant BC cells was decreased by doxorubicin (doxorubicin concentration: half of IC50 value of doxorubicin-resistant BC cells). *p<0.05, **p<0.01. MCF-7-DoxR, doxorubicin-resistant MCF-7; MDA-MB-231-DoxR, doxorubicin-resistant MDA-MB-231; Dox, doxorubicin; OD, optical density.

**Figure S3 Effect of doxorubicin on the apoptosis of doxorubicin-resistant MCF-7 cells and MCF-7 cells.** A The apoptosis of doxorubicin-resistant MCF-7 cells by doxorubicin was less than that of MCF-7 cells (doxorubicin concentration: half of IC50 value of MCF-7 cells). B The apoptosis of doxorubicin-resistant MCF-7 cells was increased by doxorubicin (doxorubicin concentration: half of IC50 value of doxorubicin-resistant MCF-7 cells). C The expression of cleaved PARP and cleaved caspase-3 in MCF-7 cells. **p<0.01. MCF-7-DoxR, doxorubicin-resistant MCF-7; Dox, doxorubicin; PI, propidium iodide; PARP, poly (ADP-ribose) polymerase.

**Figure S4 Effect of doxorubicin on the apoptosis of doxorubicin-resistant MDA-MB-231 cells and MDA-MB-231 cells.** A The apoptosis of doxorubicin-resistant MDA-MB-231 cells by doxorubicin was less than that of MDA-MB-231 cells (doxorubicin concentration: half of IC50 value of MDA-MB-231 cells). B The apoptosis of doxorubicin-resistant MDA-MB-231 cells was increased by doxorubicin (doxorubicin concentration: half of IC50 value of doxorubicin-resistant MDA-MB-231 cells). C The expression of cleaved PARP and cleaved caspase-3 in MDA-MB-231 cells. **p<0.01. MDA-MB-231-DoxR, doxorubicin-resistant MDA-MB-231; Dox, doxorubicin; PI, propidium iodide; PARP, poly (ADP-ribose) polymerase.

**Figure S5 Effect of FTO siRNAs and FTO overexpression plasmids on the FTO expression in doxorubicin-resistant BC cells and their parental cells.** A, B The FTO protein expression in BC cells and their parental cells. C, D The FTO mRNA expression in BC cells and their parental cells. **p<0.01. MCF-7-DoxR, doxorubicin-resistant MCF-7; MDA-MB-231-DoxR, doxorubicin-resistant MDA-MB-231; siNC, negative control siRNA; siFTO, FTO siRNA; NC-OE, empty vector; OE-FTO, FTO overexpression.

**Figure S6 Effect of STAT3 siRNAs and FTO overexpression plasmids on the p-STAT3 and STAT3 expression in doxorubicin-resistant BC cells.** MCF-7-DoxR, doxorubicin-resistant MCF-7; MDA-MB-231-DoxR, doxorubicin-resistant MDA-MB-231; siNC, negative control siRNA; siSTAT3, STAT3 siRNA; NC-OE, empty vector; OE-FTO, FTO overexpression; p-STAT3, phosopho-STAT3^Tyr705^.

**Figure S7 The inhibition rate of Hs578T cells by doxorubicin.**
