# Supplementary figures and images for: Fat mass and obesity-associated protein (FTO) mediates signal transducer and activator of transcription 3 (STAT3)-drived resistance of breast cancer to doxorubicin
Source: Bioengineered. 2021 Jun 2;12(1):1874–89. doi: 10.1080/21655979.2021.1924544 (PMC8806322; doi:10.1080/21655979.2021.1924544)

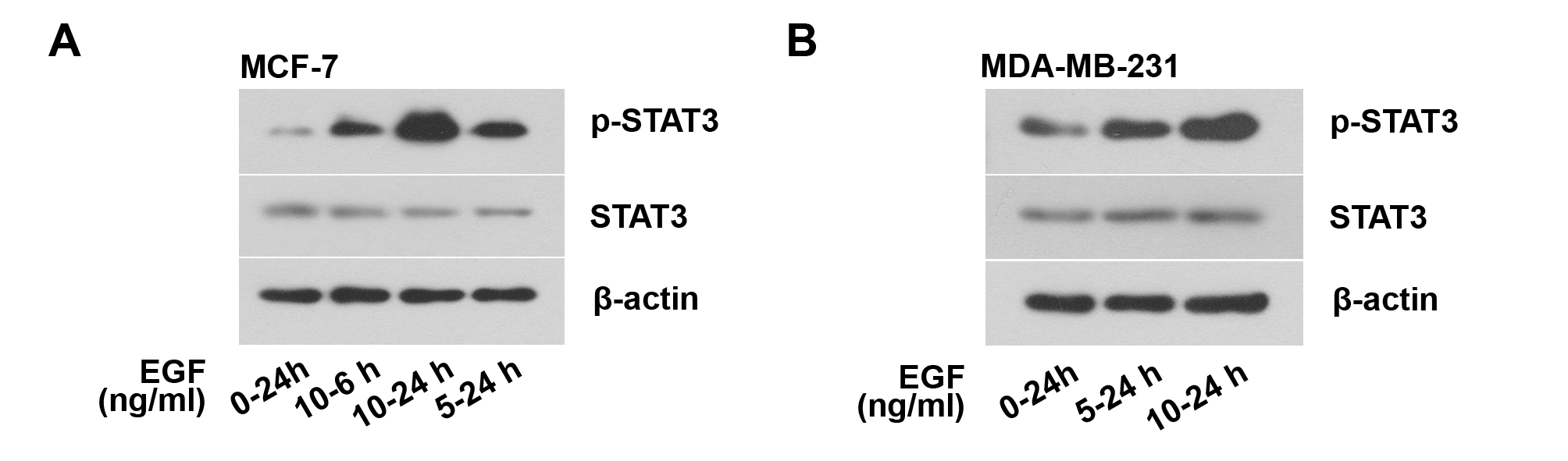

Supplement: Supplemental Material [file KBIE_A_1924544_SM7492.zip › Fig S1.tif]

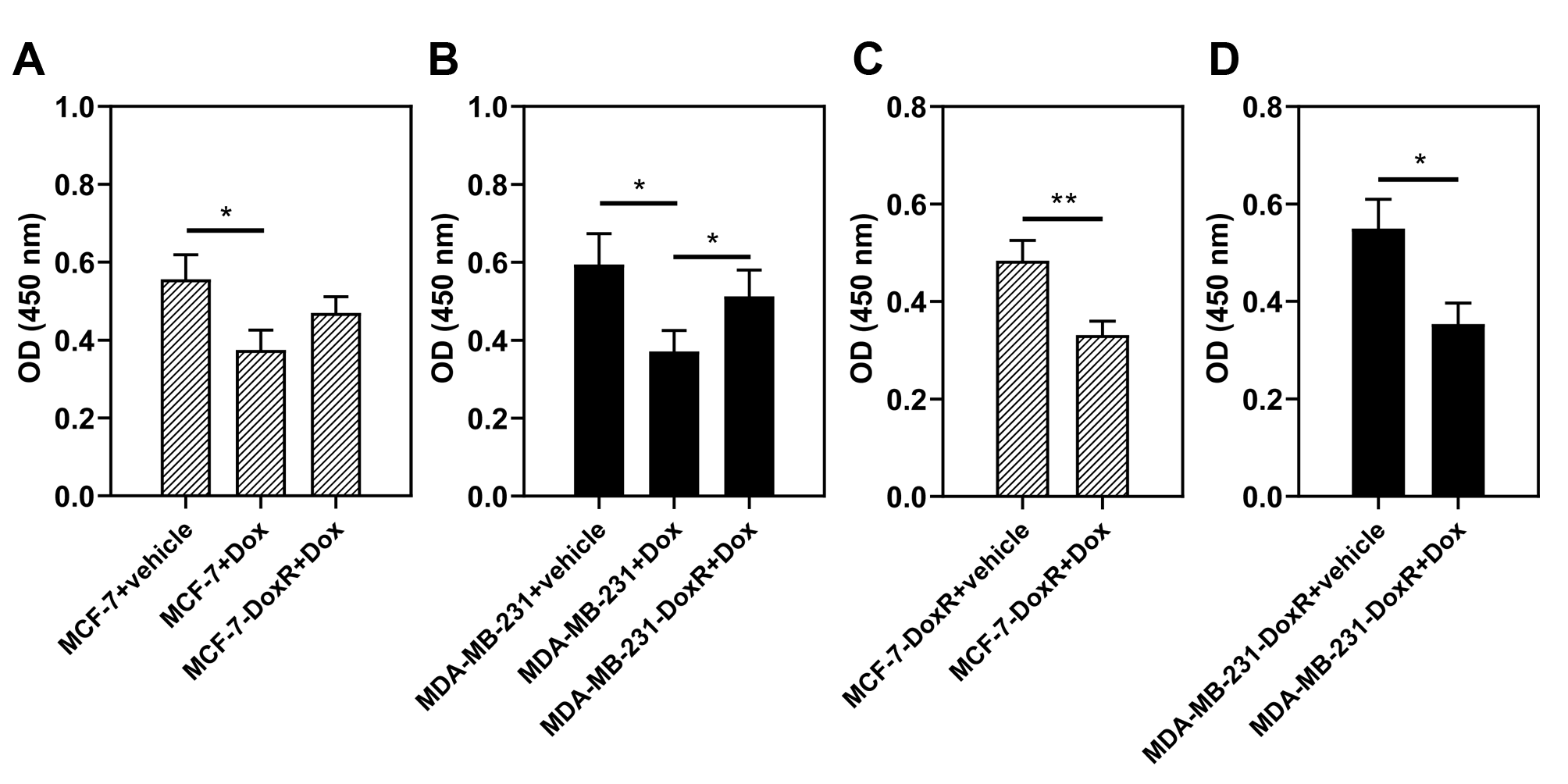

Supplement: Supplemental Material [file KBIE_A_1924544_SM7492.zip › Fig S2.tif]

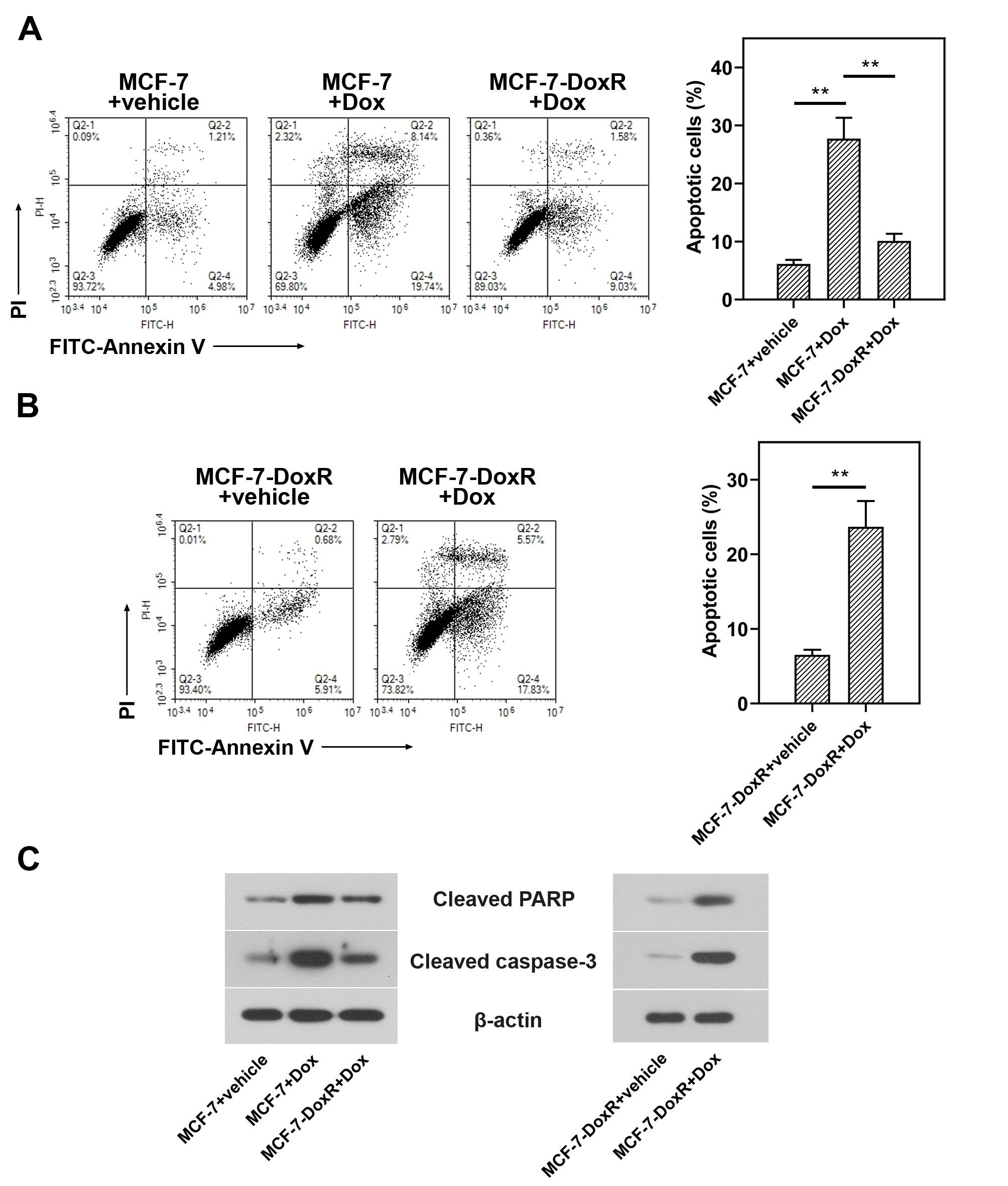

Supplement: Supplemental Material [file KBIE_A_1924544_SM7492.zip › Fig S3.tif]

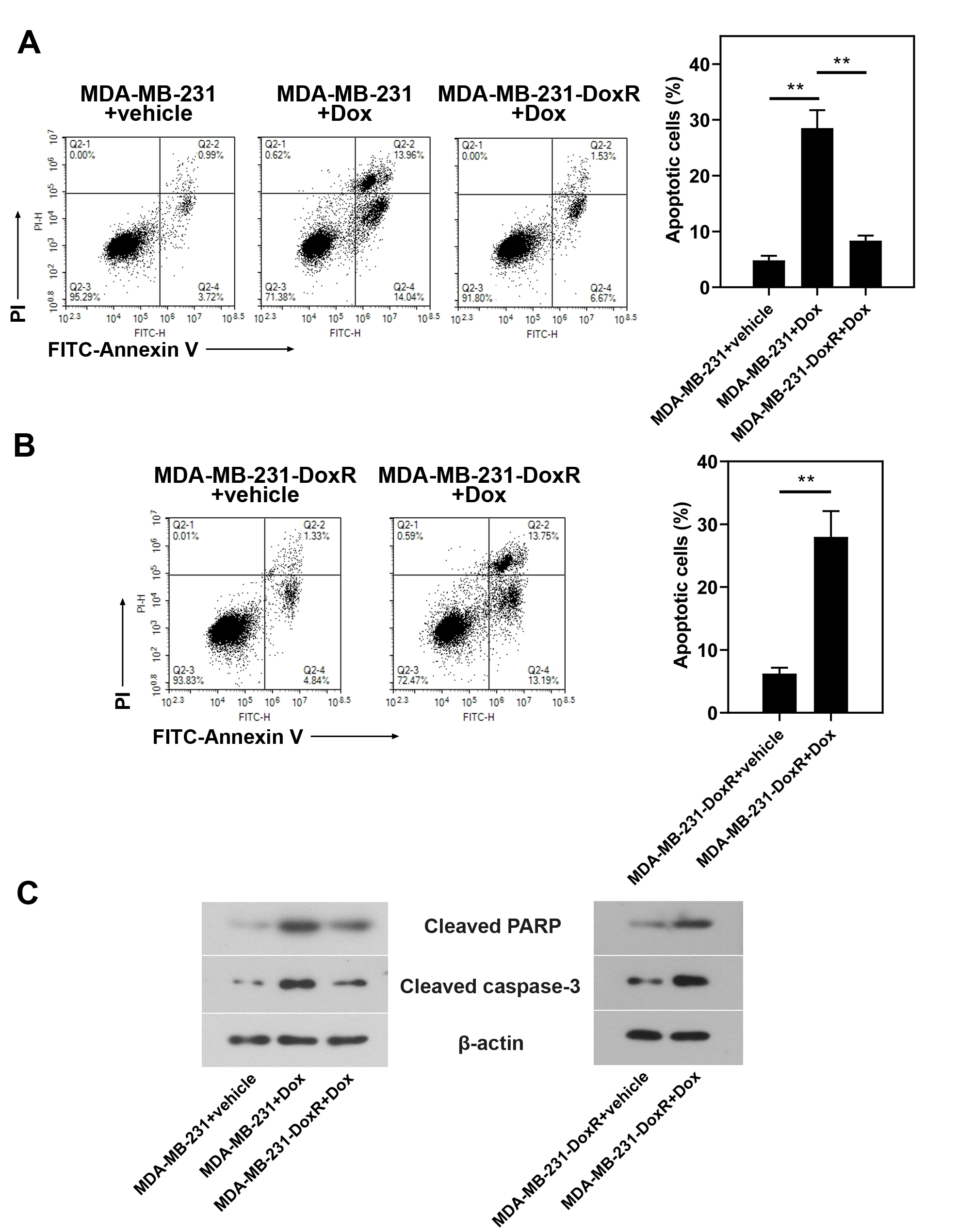

Supplement: Supplemental Material [file KBIE_A_1924544_SM7492.zip › Fig S4.tif]

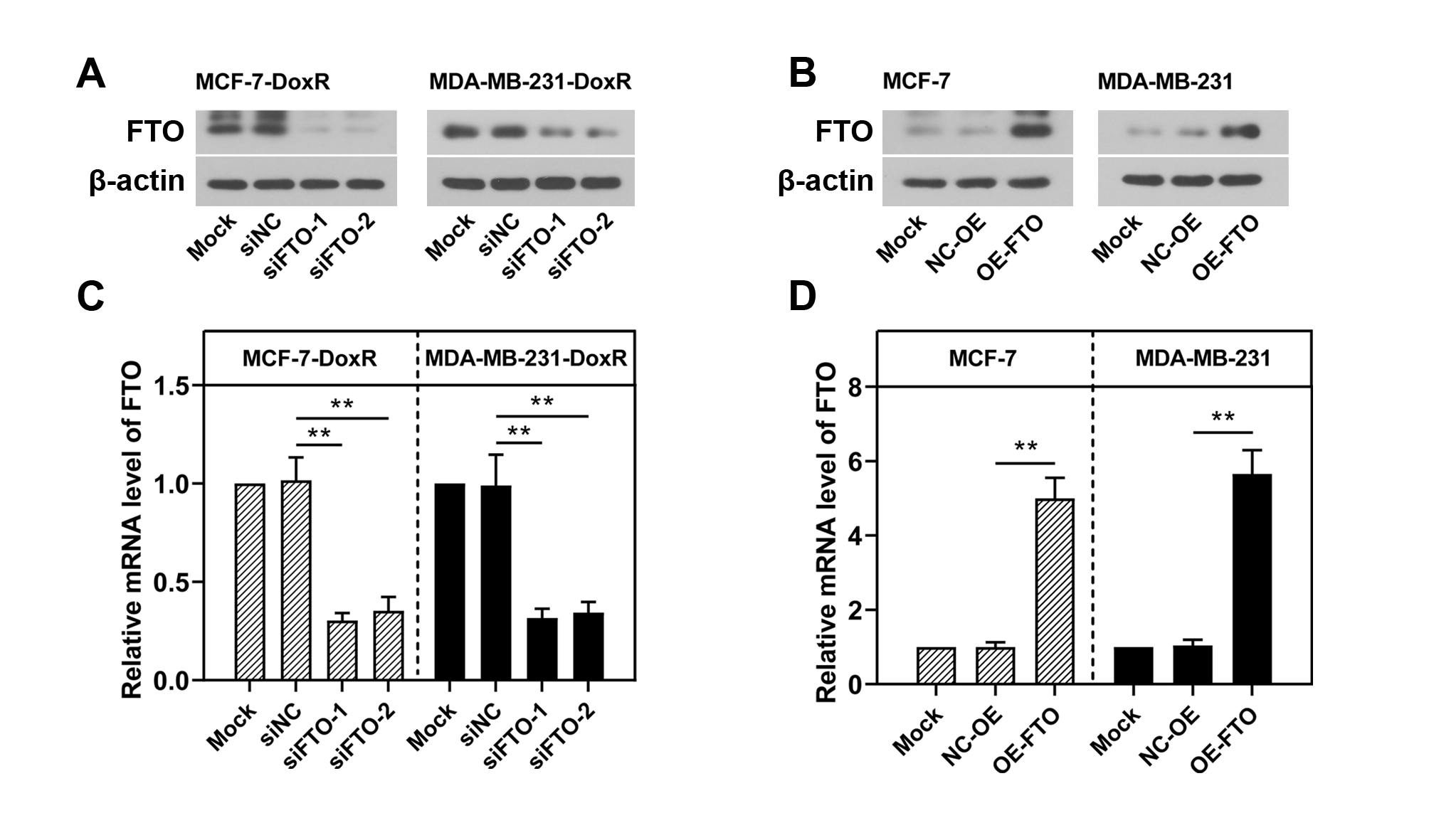

Supplement: Supplemental Material [file KBIE_A_1924544_SM7492.zip › Fig S5.tif]

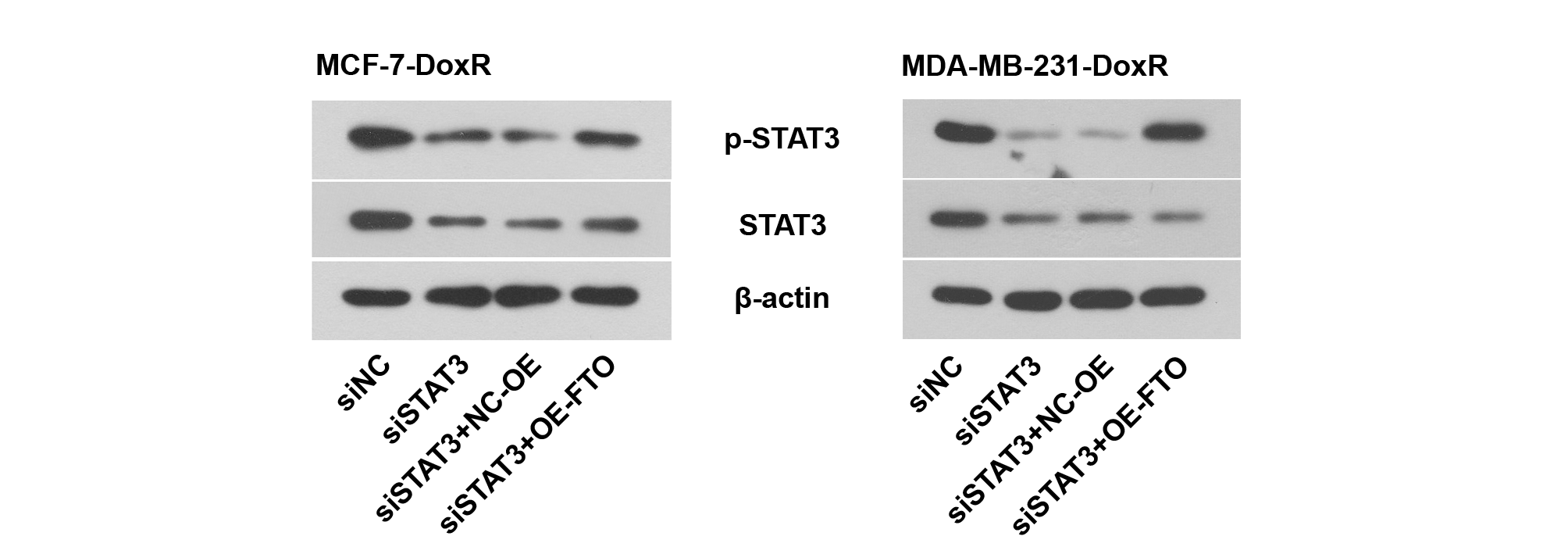

Supplement: Supplemental Material [file KBIE_A_1924544_SM7492.zip › Fig S6.tif]

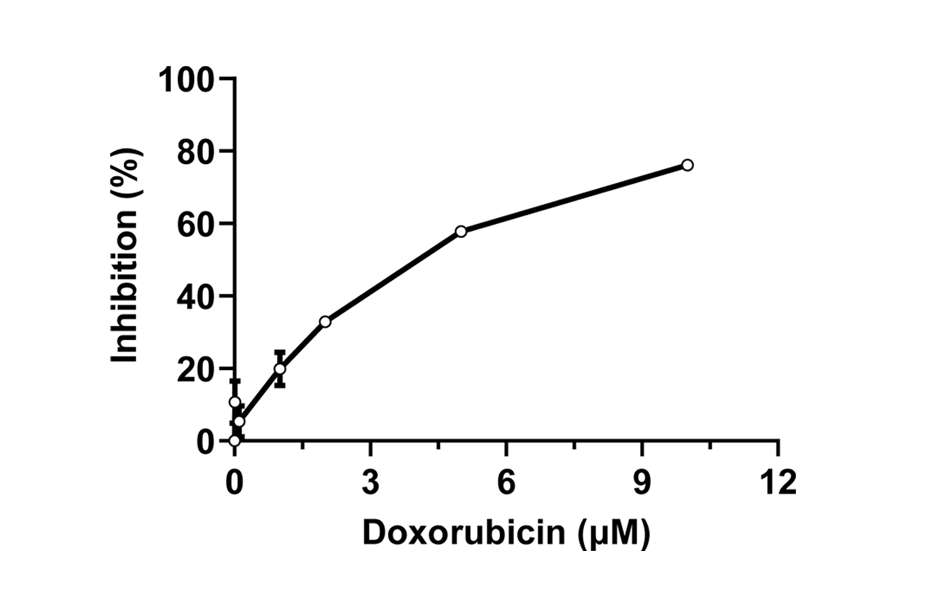

Supplement: Supplemental Material [file KBIE_A_1924544_SM7492.zip › Fig S7.tif]
